# Magnetic Molecularly Imprinted Chitosan Combined with a Paper-Based Analytical Device for the Smartphone Discrimination of Tryptophan Enantiomers

**DOI:** 10.3390/bios13080830

**Published:** 2023-08-20

**Authors:** Abdelhafid Karrat, Juan José García-Guzmán, José María Palacios-Santander, Aziz Amine, Laura Cubillana-Aguilera

**Affiliations:** 1 Department of Analytical Chemistry, Institute of Research on Electron Microscopy and Materials (IMEYMAT), Faculty of Sciences, Campus de Excelencia Internacional del Mar (CEIMAR), University of Cadiz, Campus Universitario de Puerto Real, Polígono del Río San Pedro S/N, 11510 Puerto Real, Cádiz, Spain; hafidkarrat@gmail.com (A.K.); laura.cubillana@uca.es (L.C.-A.); 2 Laboratory of Process Engineering & Environment, Faculty of Science and Technology, Hassan II University of Casablanca, B.P. 146, Mohammedia 28810, Morocco

**Keywords:** chitosan, magnetic molecularly imprinted polymer, discrimination of enantiomers, tryptophan, smartphone, paper-based analytical device

## Abstract

The separation of enantiomers plays a critical role in pharmaceutical development, ensuring therapeutic efficacy, safety, and patent protection. It enables the production of enantiopure drugs and enhances our understanding of the properties of chiral compounds. In this study, a straightforward and effective chiral detection strategy was developed for distinguishing between tryptophan (TRP) enantiomers. The approach involved the preparation of a magnetic molecularly imprinted chitosan (MMIC) for preparation of the sample, which was combined with a nitrocellulose membrane (a paper-based analytical device, PAD) integrated with D-TRP covalently grafted with polymethacrylic acid (PAD-PMA_D-TRP). Discriminating between the TRP enantiomers was achieved using AuNPs as a colorimetric probe. Indeed, the presence of D-TRP rapidly induced the aggregation of AuNPs due to its strong affinity to PAD-PMA_D-TRP, resulting in a noticeable change in the color of the AuNPs from red to purple. On the other hand, L-TRP did not induce any color changes. The chiral analysis could be easily performed with the naked eye and/or a smartphone. The developed method exhibited a detection limit of 3.3 µM, and it was successfully applied to detect TRP in serum samples, demonstrating good recovery rates. The proposed procedure is characterized by its simplicity, cost-effectiveness, rapidity, and ease of operation.

## 1. Introduction

The majority of drugs, pharmaceuticals, and biologically active compounds are mixtures of chiral isomers, which have similar chemical and physical properties. However, in most cases, one isomer exhibits the desired biological and toxicological effects, while the other may be inactive or have different pharmacological effects [1,2]. For instance, the tragic incident in the early 1960s involving thalidomide revealed that only the (R)-enantiomer provided pain relief, while the (S)-enantiomer caused severe deformities in unborn children [3]. Another example is TRP, which has two enantiomers with distinct activities [4,5]. L-TRP is a vital component of proteins and is a precursor to melatonin and serotonin, which aids in sleep and improvements in mental health. On the other hand, D-TRP, a non-protein amino acid, does not participate in the metabolic pathways of living systems but it is commonly used in the synthesis of immunosuppressant and peptide antibiotics [6,7,8]. The importance of chiral molecules in the production of pharmaceutical and biologically active compounds has led to a significant demand for effective separation techniques to purify chiral molecules from racemic mixtures. Molecular imprinting is a promising method of separation, as it relies on the spatial structure of the target molecules [9,10].

Molecularly imprinted polymers (MIPs) are cross-linked polymers formed through polymerization of a functional monomer (or co-monomers) in the presence of a template and a crosslinking agent [11,12]. Once the template is removed, recognition cavities are created within the polymer, which selectively recognize the target molecule in a mixture of compounds [13,14]. MIPs have found wide applications for various analytes such as viruses, bacteria, emerging pollutants, pharmaceutical drugs, and pesticides [15,16,17]. MIPs are largely used for the preparation of samples and the extraction of target analytes from complex matrices when combined with magnetic nanoparticles (MNPs) [18,19,20]. This combination offers several advantages. Firstly, it enables selective extraction of the target analytes from complex samples. Secondly, the magnetic properties of MNPs allow efficient and rapid extraction, as they can be easily manipulated and separated using a magnet. Additionally, MIPs provide a robust and reusable platform for selective extraction, as they can be regenerated and reused multiple times [21,22].

Chitosan, a polysaccharide derived from chitin, is particularly suitable for the development of MIPs due to its non-toxic, biocompatible, bioactive, and biodegradable properties. Its abundance of amino and hydroxyl groups allows it to react with various cross-linking agents for the preparation of MIPs [23,24,25]. In recent years, the use of gold nanoparticles (AuNPs) as optical labels has resulted in the development of numerous sensors and biosensors [26,27,28]. The simplicity of the synthesis of AuNPs and its intense red color, visible to the naked eye, make it a popular choice. However, research on colorimetric chiral discrimination using metal nanoparticles is limited, and a straightforward device for this purpose is yet to be developed. Consequently, constructing a reliable, user-friendly, sensitive, and high-throughput assay for determining the enantiomers of chiral substances still remains a challenge.

A PAD is a low-cost, portable, and disposable device that utilizes paper as the primary substrate for performing analytical tests. It integrates various components, such as sample application zones and detection zones for performing chemical or biological assays. The concept behind PADs is to leverage the properties of paper, such as its capillary action and porous structure, to facilitate fluid flow and reaction processes. The design typically involves creating channels on the paper’s surface to direct the movement of the liquid samples and reagents [29,30]. PADs can be used for a wide range of applications, including clinical diagnostics, environmental monitoring, food safety testing, and drug detection. They often require minimal or no instrumentation, making them particularly suitable for resource-limited settings and point-of-need testing [31].

In this work, a magnetic molecularly imprinted chitosan (MMIC) was developed for the extraction of TRP and combined with a PAD for to discriminate between the enantiomers of TRP. The proposed PAD was based on a nitrocellulose membrane modified with D-TRP-grafted polymethacrylic acid (PMA_D-TRP), successfully leading to good chiral separation using AuNPs as a colorimetric probe. The developed procedure holds promise for applications in pharmaceutical analysis, offering a reliable and user-friendly approach for chiral analysis.

## 2. Materials and Methods

### 2.1. Material and Apparatus

Chitosan, FeCl_3_·6H_2_O, FeCl_2_·4H_2_O sulfuric acid (H_2_SO_4_), L-TRP, D-TRP, methacrylic acid, ethylene glycol dimethylacrylate (EGDMA), azobisisobutyronitrile (AIBN), dimethylsulfoxide (DMSO), copper (II) chloride (CuCl_2_), acetic acid (AcH), polyphosphoric acid (H_3_PO_4_), and boric acid (H_3_BO_3_) were purchased from Sigma-Aldrich (Steinheim, Germany). Membrane filters (nitrocellulose, 0.45 µm, 47 mm in diameter) were purchased from Sigma-Aldrich.

Scanning/transmission electron microscopy (TEM) images were acquired using FEI Nova NANOSEM 450 equipment (Thermo Fisher Scientific, Waltham, MA, USA) (resolution = 1 nm). Fourier transform infrared (FT-IR) spectra were collected using aIR Affinity-1S spectrophotometer (Shimadzu, Japan) in the attenuated total reflectance (ATR) mode and across the range of 4000–500 cm^−^^1^. The UV absorption spectra were measured by a double beam UV/vis spectrophotometer (model T80+, PG Instruments, Wibtoft, Leicestershire, UK). The pictures for the discrimination of TRP were taken with an Android smartphone with a 13 MP camera. Nanopure water was prepared by a Wasser Lab Ultramatic Plus (Type I) system (Barbatáin, Navarra, Spain) and used in all experiments.

### 2.2. Synthesis of the Magnetite Nanoparticles

The synthesis of Fe_3_O_4_ nanoparticles was carried out using the method described in [32]. In brief, a three-necked flask was prepared under a N_2_ atmosphere, and a solution containing 13.56 g of FeCl_3_·6H_2_O and 4.96 g of FeCl_2_·4H_2_O dissolved in 250 mL of distilled water was prepared. Subsequently, 20 mL of ammonium hydroxide was introduced into the flask, and the mixture was vigorously stirred for 40 min at 80 °C. The resulting Fe_3_O_4_ nanoparticles were then collected using an external magnet and washed with distilled water to eliminate any residual chemicals. The obtained precipitate was finally dried at 50 °C in a vacuum oven.

### 2.3. Synthesis of Molecularly Imprinted Chitosan

The method of preparing MMIC was as follows. Initially, 100.0 mg of chitosan was dissolved in 10.0 mL of a 1.0% (*v*/*v*) aqueous acetic acid solution. At the same time, 6.0 mg of the TRP template molecules was added into the chitosan solution, followed by the addition of 100 mg of Fe_3_O_4_ and stirred at room temperature for 4 h. Subsequently, 0.5 M sulfuric acid was added to the solution and stirred for 2 h. After that, the TRP template molecules were removed by an ethanol solvent. A magnetic non-imprinted chitosan (MNIC) was prepared following the same procedure, except for the addition of the template.

### 2.4. Synthesis of Gold Nanoparticles

Gold nanoparticles were synthesized according to our previous study [33]. Initially, a cylindrical glass vessel was placed in a water bath containing 1.25 mL of a 1.0 mM HAuCl_4_ aqueous solution. To ensure a consistent temperature, the sample vessel remained in the water bath at an ambient temperature throughout the process, as the local heating from sonication affected the solution’s temperature. After 4 min of sonication, 250 µL of an aqueous solution of 38.8 mM sodium citrate dihydrate was added to the vessel. This addition caused an immediate change in the solution’s color to grey. Continuous sonication led to a subsequent transformation of the solution’s color to dark red after 5.5 min, indicating the formation of the colloid AuNPs.

### 2.5. Synthesis of D-TRP-Grafted Polymethacrylic Acid (PMA_D-TRP)

PMA_D-TRP was prepared according to the following procedure: 20 mg of D-TRP was introduced into a beaker containing 50 mL of DMSO, followed by the addition of 700 µL of MAA and 10 mL of EGDMA. Then 10 mg of CuCl_2_ was added to link the monomer to D-TRP covalently, and 3.3 mg of AIBN was used to initiate the process of polymerization. The blend was deaerated with nitrogen for 5 min and the reaction was carried out at 60 °C for 15 min using a domestic microwave. The material thus obtained was washed and dried in oven at 60 °C.

### 2.6. Preparation of the Paper-Based Analytical Device (PAD)

For this, 10 mg of the developed material (PMA_D-TRP) was dispersed in 5 mL of nanopure water and filtrated under a vacuum to entrap the PMA_D-TRP in the nitrocellulose membrane’s pores (porosity: 0.45 µm). After filtration, the paper was dried in an oven for 15 min at 40 °C. The paper was then cut into small disks for further use and named PAD-PMA_D-TRP.

### 2.7. Adsorption Study

For this study, 10 mg of MMIC/MNIC was introduced into 1 mL of D-TRP with a concentration range of 30–320 µM. The mixtures were shaken at a room temperature for 15 min. After the process of adsorption, the sorbent was separated by centrifugation at 1000 rpm for 2 min. The concentrations of the obtained supernatants were determined by the UV-spectrophotometer at a wavelength of 280 nm. The adsorption capacity (*Q*_e_) was calculated by Equation (1):

(1) $$Q=\frac{C_{i}-C_{e}}{m}V$$

### 2.8. Solid-Phase Extraction Combined with Smartphone Detection

**Scheme 1:**
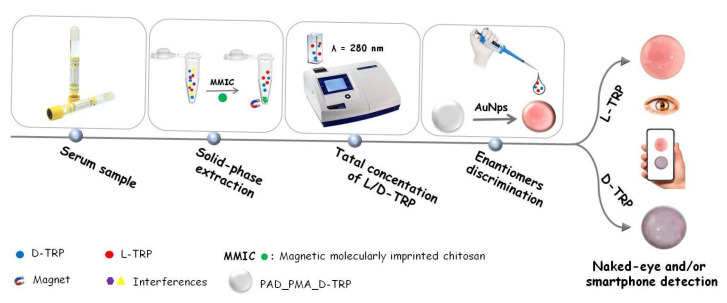
Illustration of solid-phase extraction coupled with smartphone-based discrimination of L/D-TRP enantiomers.

Solid-phase extraction is a versatile and effective technique of preparing samples that enables the selective extraction of the target analytes from complex sample matrices. In this work, the proposed MMIC was used as the sorbent in a solid-phase extraction technique for the total isolation of TRP. The task of discriminating between the TRP enantiomers was performed using the developed PAD-PMA_D-TRP. The procedure was as follows. First, 10 mg of MMIC was introduced into an Eppendorf tube containing 1 mL of a TRP solution. The blend was shaken for 30 min at room temperature. After adsorption, the TRP was eluted with 1 mL of methanol and measured by UV spectroscopy at λ = 280 nm to determine the total concentration of L/D-TRP. Then 10 µL of the supernatant was added to the developed PAD-PMA_D-TRP containing 40 µL of the dispersed AuNPs, followed by the addition of 10 µL of a Britton–Robinson (BR) buffer (0.04 M H_3_PO_4_, 0.04 M AcH, and 0.04 M H_3_BO_3_) (pH4). After 25 min, the spots were measured with a smartphone (Scheme 1).

## 3. Results and Discussion

### 3.1. Characterization Studies of Fe_3_O_4_ and MMIC

**Figure 1:**
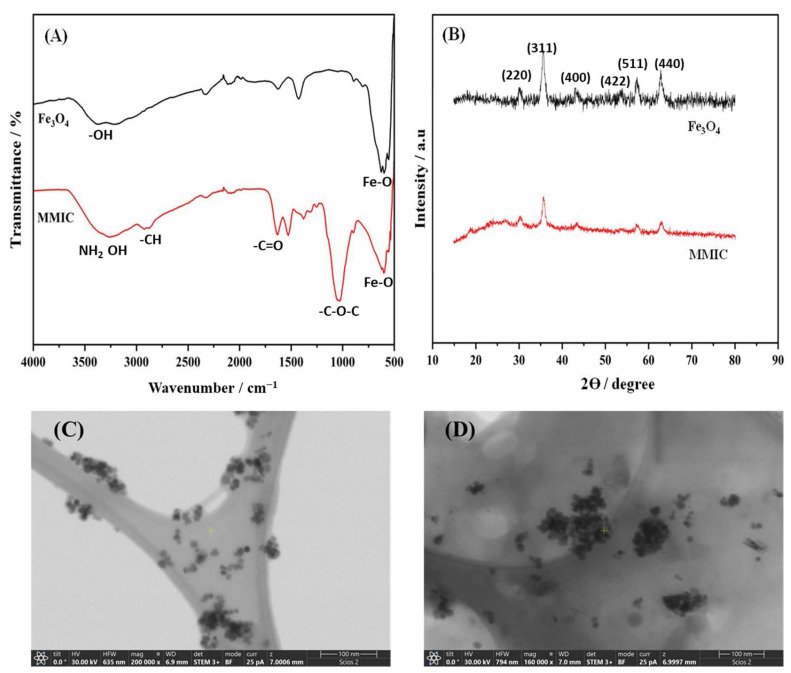
FTIR spectra (**A**) and XRD diffractograms (**B**) of Fe_3_O_4_ and MMIC. (**C**,**D**) TEM images of Fe_3_O_4_ and MMIC, respectively.

The FTIR technique was used to analyze the chemical composition and functional groups of the proposed materials (Figure 1A). The Fe_3_O_4_ typically exhibited the absorption bands of O-H stretching vibration at 3200–3600 cm^−1^ (adsorbed water) and a strong band around 550–650 cm^−1^ corresponding to the Fe-O group. However, the MMIC material presented both the characteristic chitosan absorption bands, including O-H at 3200–3600 cm^−1^, C=O 1650 cm^−1^, and N-H around 1560 cm^−1^, and the characteristic bands of Fe_3_O_4_ mentioned above. These results confirmed the modification of Fe_3_O_4_ with chitosan.

The XRD technique was also used to confirm the preparation of the materials (Figure 1B). Fe_3_O_4_ exhibits a characteristic spinel crystal structure, with peaks typically observed at 2θ values around 30°, 35°, 43°, 57°, and 62°, corresponding to the (220), (311), (400), (511), and (440) crystal planes, respectively. These peaks corresponded well with the standard XRD data of magnetic Fe_3_O_4_ (JCPDS No. 85-1436) [34]. When chitosan was decorated onto the Fe_3_O_4_, the XRD pattern showed changes in the peaks’ intensities. These changes depended on the interaction between chitosan and Fe_3_O_4_.

TEM was used to characterize the size and morphological structure of the prepared materials, namely Fe_3_O_4_ and Fe_3_O_4_–chitosan. The developed Fe_3_O_4_ particles exhibited a mean diameter of approximately 12.03 ± 3.1 nm (n = 13) (see Figure 1C). However, MMIC (Figure 1D) showed dense particles due to the presence of the polymer, which modified a large number of the Fe_3_O_4_ nanoparticles. These results strongly confirmed the successful modification of the magnetic nanoparticles with chitosan.

### 3.2. Transmission Electron Microscopy for AuNPs

**Figure 2:**
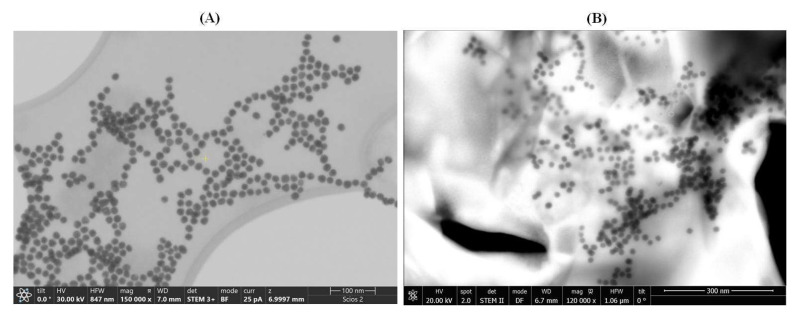
TEM images of AuNPs/L-TRP (**A**) and AuNPs/D-TRP (**B**). Micrographs were taken at different magnifications (×150,000 and ×120,000) and in brightfield (BF) and darkfield (DF) field modes.

TEM images were acquired to assess the behavior of AuNPs when exposed to D-TRP or L-TRP. As depicted in Figure 2A,B, the presence of D-TRP led to the aggregation of AuNPs. Conversely, in the presence of L-TRP, the AuNPs remained dispersed and exhibited a monodisperse distribution.

### 3.3. Adsorption Study

The adsorption study played a crucial role in the characterization of MIPs by providing insights into the binding capacity. It contributed to an understanding of the behavior of MIPs and aided in the development of cavities that were efficient and highly specific to the analyte.

**Figure 3:**
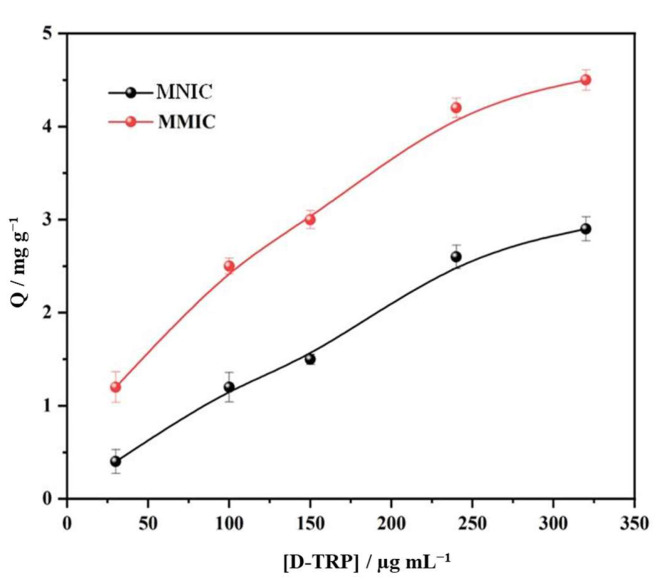
Adsorption isotherms of D-TRP on MMIC and MNIC at room temperature.

The binding isotherms of D-TRP on the MMIC and MNIC at 25  °C are presented in Figure 3. As the concentration of D-TRP increased, both the MMIC and MNIC demonstrated higher binding capacities. However, due to the presence of multiple cavities, the MMIC exhibited a higher adsorption capacity compared with the MNIC. This observation was supported by the higher values of the imprinted factor, which represents the ratio of the adsorption capacity of MMIC to MNIC. The imprinted factor values ranged between 1.5 and 2, providing further evidence for the successful development of D-TRP cavities on the surface of the MMIC.

### 3.4. Discriminating between TRP Enantiomers

**Scheme 2:**
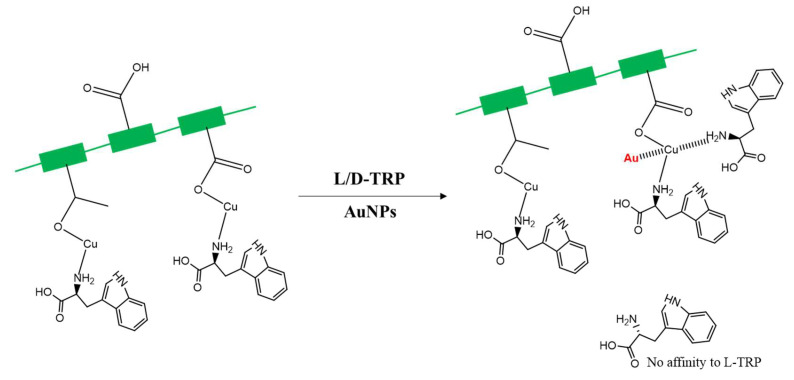
Detailed mechanism of discriminating between TRP enantiomers.

Discriminating between the two enantiomers of tryptophan is important for several reasons, including protein synthesis, the production of neurotransmitters, drug development, and understanding metabolism and health. In the present work, the discrimination was performed after the extraction of total TRP using the developed MMIC. Indeed, a paper-based analytical device was developed for discriminating between the TRP enantiomers. This device is based on a membrane modified with polymethacrylic acid grafted with D-TRP (PAD-PMA_D-TRP). For visual detection, AuNPs were used as colorimetric probes because of their sensitive aggregation with TRP. Indeed, the AuNPs changed color from red to purple through the aggregation phenomenon stimulated by TRP. The newly developed PAD-PMA_D-TRP exhibited high affinity to D-TRP, providing a rapid aggregation of AuNPs. However, there was no interaction between PAD-PMA_D-TRP and L-TRP, and thus the AuNPs maintained their red coloration (Scheme 2). This approach enabled the convenient discrimination of TRP enantiomers using a smartphone or even with the naked eye.

### 3.5. Smartphone Detection

To enable on-site quantitative evaluation of the color change exhibited by AuNPs in the presence of TRP, we combined our PAD-PMA_D-TRP with a smartphone. This allowed us to monitor alterations in the RGB values as the red color of the AuNPs transitioned to a purplish-blue upon the addition of TRP. The standard RGB scale uses whole-number values ranging from 0 to 255 to represent the intensity of each color channel: red, green, and blue. In this scale, [255, 255, 255] corresponds to pure white, while [0, 0, 0] represents absolute black [35,36,37,38,39]. By utilizing smartphone-based RGB detection, we were able to observe and record the changes in color. The RGB values were measured by Image J software 1.53t, and the difference between the red and blue (R-B) values was plotted against the concentration of TRP.

### 3.6. Optimization of the Detection Procedure

The volume of AuNPs, the pH conditions, and the time of color developement are the most important parameters to be optimized in order to achieve an effective means of discriminating between TRP enantiomers.

**Figure 4:**
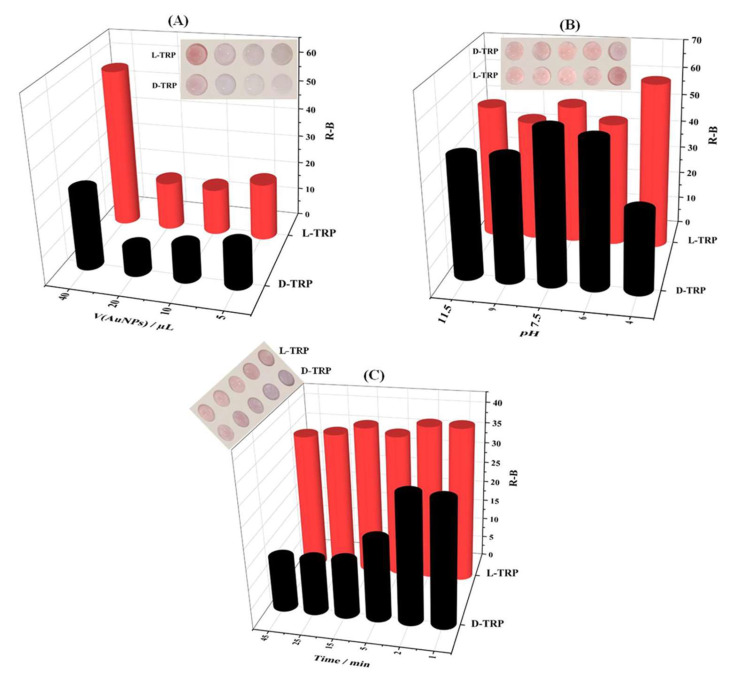
Effect of different parameters on the optimal discrimination of TRP enantiomers including (**A**) the volume of AuNPs (pH 4 and *t* = 25 min), (**B**) the optimal pH (AuNPs = 40 µL and *t* = 25 min), and (**C**) the time of color development (AuNPs = 40 µL, pH 4).

As mentioned above, AuNPs were used as the colorimetric probe for the discrimination of TRP. Thus, the volume of AuNPs is a relevant parameter to be optimized. Figure 4A shows that 40 µL of AuNPs was the most suitable volume to be added to the PAD-PMA_D-TRP for the colorimetric discrimination of L-TRP and D-TRP enantiomers.

The aggregation of AuNPs depends on the pH of the solution (Figure 4B). Indeed, the pH effect of the BR buffer was studied in the pH range of 4–11.5. A good interaction between D-TRP and AuNPs was achieved at a pH value of 4. However, the L-TRP had no effect on the AuNPs’ coloration. Furthermore, the effect of time on the ability to discriminate between the TRP enantiomers was also examined (Figure 4C). The addition of D-TRP showed a significant decrease in the R-B intensity compared with L-TRP. A significant difference between L-TRP and D-TRP was obtained at 25 min. Therefore, this time was utilized for subsequent experiments.

### 3.7. Calibration Curve

**Figure 5:**
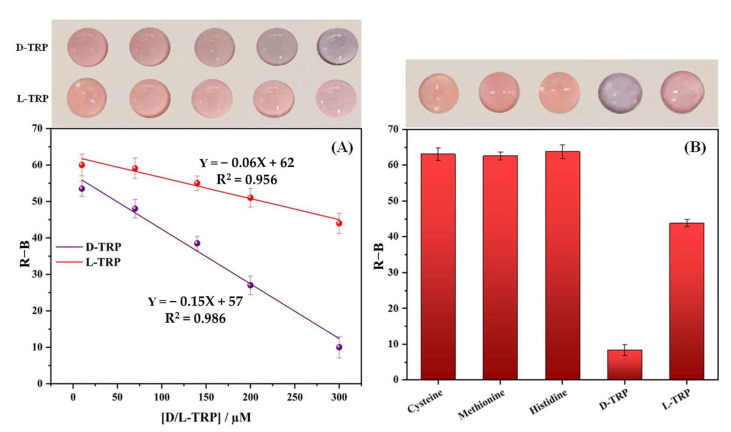
The change in color intensity (R-B) as a function of the concentration of D- and L-TRP between 10 and 300 µM for the optimized assay (**A**). Selectivity of the developed PAD-PMA_D-TRP device (**B**) (n = 3).

Figure 5A shows the calibration curves of D-TRP and L-TRP obtained using different concentrations ranging from 10 to 300 µM. As shown in the photographs, the color of the PAD-PMA_D-TRP color changed from pink to purple, and became more intense with an increasing concentration of D-TRP; thus, aggregation of the AuNPs occurred. However, with the addition of L-TRP, the PAD-PMA_D-TRP showed no significant color change, indicating the low interaction between L-TRP and the developed material. To quantitatively evaluate the results, the color density was converted into the R-B color intensity. This relationship can be described by the equation R-B = −0.15X + 57, where X represents the concentration of the analyte. The correlation coefficient for this linear relationship was 0.986, indicating a strong association. The limit of detection (LOD) for D-TRP was determined to be 3.3 µM, whereas the LOD for L-TRP could not be calculated due to the absence of a significant color change. These findings were based on three individual assays (*n* = 3).

### 3.8. Selectivity Study

A selectivity study plays a pivotal role in ensuring accurate and reliable analyses by identifying the target analytes and eliminating interference. The response of the newly developed PAD-PMA_D-TRP was tested with other amino acids, including cysteine, methionine, histidine, and L-TRP. Figure 5B presents the results obtained under the optimal conditions. It is evident that only D-TRP exhibited a lower R-B response compared with the other amino acids. Notably, L-cysteine was found to cause aggregation of the AuNPs. However, the use of the MMIC in the sample’s preparation effectively removed interferences such as L-cysteine thank to its specific cavities, which were complementary only to TRP in terms of the shape, size, and functional groups. By combining the MMIC with the newly developed PAD-PMA_D-TRP, successful discrimination of TRP enantiomers was achieved.

### 3.9. Enantioselective Measurement of L/D Tryptophan Mixtures

The primary objective was to investigate whether the discriminative sensing capability of AuNPs could be utilized for determining the enantiomeric percentage using the PAD-PMA_D-TRP. Since TRP commonly exists as enantiomeric pairs, it is crucial to assess the influence of one enantiomer on the other. With the proposed approach, we were able to directly evaluate the performance of the PAD-PMA_D-TRP in determining the percentage and confirming the enantioselective separation and purification of TRP in a racemic solution using AuNPs. Indeed, after the preparation of the samples using the developed MMIC, the concentration of L/D-TRP in the resulting supernatant could be determined by measuring the total concentration at 280 nm using UV spectroscopy. Subsequently, the percentage of each enantiomer was determined using the PAD-PMA_D-TRP.

**Figure 6:**
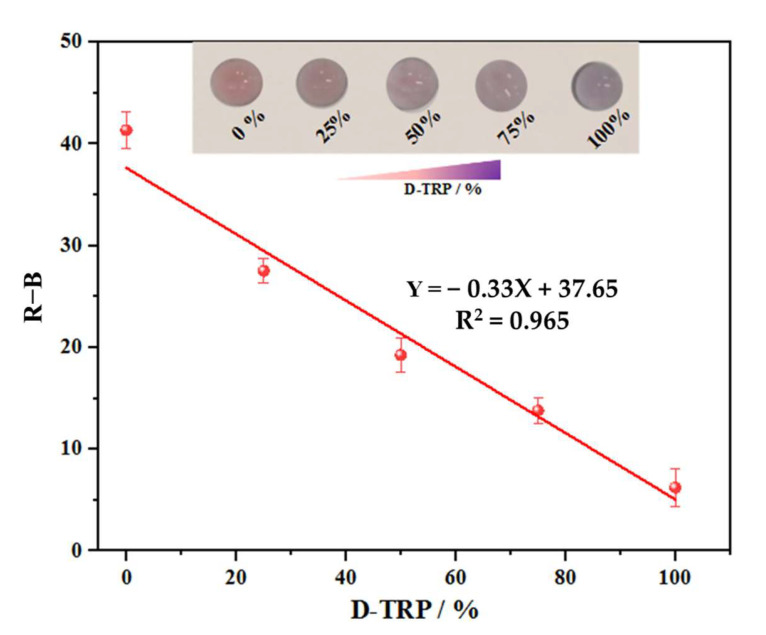
The linear decrease in the R-B response as a function of D-TRP (%) in the range from 0% to 100%. The total concentration of L/D-TRP was 300 µM.

An interesting observation is related to the change in color from red to purple, which is dependent on the enantiomeric ratio. Figure 6 illustrates that the aggregation of AuNPs was selectively induced by D-TRP, leading to the precipitation of D-TRP with AuNPs. As a result, the excess of the other enantiomer remained in the solution, thereby enabling enantioseparation. The graph in Figure 6 demonstrates a linear decrease in the R-B intensity with an increasing enantiomeric percentage of D-TRP, ranging from 0% to 100%, indicating the optimized assay’s ability to quantify the enantiomeric composition accurately.

### 3.10. Real Sample

With the aim of using the proposed PAD-PMA_D-TRP for a biological sample, it was utilized for the determination of D-TRP in a human serum sample. Samples spiked with 30 and 100 µM of D-TRP were tested. The results are presented in Table 1 along with satisfactory recoveries of 88.6–106.4%. The obtained results confirmed the applicability of the developed device for the detection of D-TRP in real biological samples.

## 4. Conclusions

The discrimination of enantiomers is necessary in order to understand their distinct biological activities, ensure the safety and efficacy of chiral drugs, and achieve analytical accuracy. This study presents a straightforward approach for the chirally selective sensing of TRP enantiomers using AuNPs as a colorimetric probe. The combination of MMIC with the developed PAD-PMA_D-TRP enabled the chiral recognition of TRP. The detection of TRP enantiomers could be easily read with the naked eye and/or a smartphone. Remarkably, the proposed sensor demonstrated the capability to detect D-TRP in human serum, suggesting its potential as a valuable platform for analyzing real samples. This paper-based device represents the first example of a simple, cost-effective, and user-friendly platform for enantioselective sensing applications. The success of this application opens up new possibilities for designing innovative enantiosensing strategies in the future.

## Figures and Tables

**Table 1:** The application of the proposed method for the determination of D-TRP in a spiked human serum sample.

Added (µM)	Found (µM)	Recovery (%)	RSD (%) *
30	26.5	88.6	3.4
100	106.4	106.4	2.7

* n = 3.

## Data Availability

The data used to support the findings of this study are available from the corresponding author upon request.
